# Long term nutritional outcome of children fed an amino-acid formula

**DOI:** 10.1186/2045-7022-3-S3-P16

**Published:** 2013-07-25

**Authors:** D Colson, B Michaud, P Soulaines, C Dupont

**Affiliations:** 1 Nutritia Nutrition Clinique, Saint Ouen, France; 2 Laboratoire d’Immunologie Biologie INSERM U1013, Paris, France; 3 Hopital Necker Enfants-Malades, Paris, France

## Background

Some cases of cow’s milk protein allergy (CMPA) require the use of an amino-acids based formula (AAF), with a with a protein level higher than in standard infant formulas. This study assessed the long term consequences of an AAF-based elimination diet on the clinical and biological outcome of CMPA children.

## Methods

Retrospective analysis of consecutive (2004-2010) patients diagnosed with CMPA (digestive/cutaneous/respiratory symptoms) in a food allergy reference center (Cohort Arsène,n°DC-2009-955), through a telephone survey (Nov2011–Feb2012). Children were enrolled if having adhered to dietetic recommendations, and fed with ≥500mL/day of AAF (Neocate®) or extensive whey hydrolysate-based formula (eWHF, Pepti-Junior®) for ≥6mo, starting either before age 6 months (AAF1, eWHF1)), between ages >6 and 12 months (AAF2) or after age 12 (AAF3).

## Results

From a longitudinal data set of 515 children, 134 responded to enrolment criteria, 102 fed with AAF and 32 fed with eWHF. At survey, BMI (percentiles) in males was in the normal range and identical whether fed by AAF or eWHF. In females, BMI was significantly lower in AAF1vs eWHF1 (p 0.04). Ferritin and hemoglobin levels were within the normal range in all groups.

**Table 1:** 

Age at survey (months) (Mean, SD)	AAF1(n=41)66.02±26.5	eHWF1(n=20)86.55±24	AAF2(n=42)62.4±34.3	AAF3(n=20)73.5±28.7
BMI percentile in males at survey: (Mean, SD)	42.85±28.79	44.17±30.07	42.73±29.37	31±25.09

BMI percentile in females at survey (Mean, SD)	31.91±38	64.16±26.07	31.54±24.95	33.85±22.68

Ferritin in males/females (Mean, SD)	26.7±13.6/27±17	11.98±0.8/12.6±0.4	11.64±0.6/12.7±0.4	30.6±14.1/26.5±0.5

Hemoglobin in males/females (Mean, SD)	31.7±9.8/35±8.6	11.05±0.55/35.12±30.3	12.38±0.75/11.6±0.6	34.3±12.28/11.36±1.8

## Conclusion

This study suggests that feeding CMPA children with>500 mL/day of AAF for >15 mo showed a good nutritional outcome with an appropriate anthropomorphic parameters and iron status.

## Disclosure of interest

D Colson: Grant/research support from Nutricia Nutrition Clinique, B Michaud: None declared, P Soulaines: None declared, C Dupont: None declared.

